# Type 1 early infantile epileptic encephalopathy: A case report and literature review

**DOI:** 10.1002/mgg3.2412

**Published:** 2024-02-23

**Authors:** Erfan Zaker, Negar Nouri, Mojtaba Movahedinia, Ali Dadbinpour, Mohammad Yahya Vahidi Mehrjardi

**Affiliations:** ^1^ Department of Medical Genetics, Faculty of Medicine Shahid Sadoughi University of Medical Sciences Yazd Iran; ^2^ Department of Children Growth Disorder Research Center Shahid Sadoughi University of Medical Sciences Yazd Iran; ^3^ Department of Medical Genetics School of Medicine Shahid Sadoughi University of Medical Sciences Yazd Iran; ^4^ Diabetes Research Center Shahid Sadoughi University of Medical Sciences Yazd Iran

**Keywords:** Aristaless‐related homeobox, *ARX*, early infantile epileptic encephalopathy, EIEE1, Ohtahara syndrome

## Abstract

**Background:**

Variants in the Aristaless‐related homeobox (*ARX*) gene lead to a variety of phenotypes, with intellectual disability being a steady feature. Other features can include severe epilepsy, spasticity, movement disorders, hydranencephaly, and ambiguous genitalia in males. X‐linked Ohtahara syndrome or Type 1 early infantile epileptic encephalopathy (EIEE1) is a severe early‐onset epileptic encephalopathy with arrested psychomotor development caused by hemizygous mutations in the *ARX* gene, which encodes a transcription factor in fundamental brain developmental processes.

**Methods:**

We presented a case report of a 2‐year‐old boy who exhibited symptoms such as microcephaly, seizures, and severe multifocal epileptic abnormalities, and genetic techniques such as autozygosity mapping, Sanger sequencing, and whole‐exome sequencing.

**Results:**

We confirmed that the patient had the NM_139058.3:c.84C>A; p.(Cys28Ter) mutation in the *ARX* gene.

**Conclusion:**

The patient with EIEE1 had physical symptoms and hypsarrhythmia on electroencephalogram. Genetic testing identified a causative mutation in the *ARX* gene, emphasizing the role of genetic testing in EIEE diagnosis.

## Graphical abstract

Variants in Aristaless‐related homeobox (ARX) gene lead to a variety of phenotypes with intellectual disability being a steady feature. Other features can include severe epilepsy, spasticity, movement disorders, hydranencephaly, and ambiguous genitalia in males. Ohtahara syndrome or Type 1 early infantile epileptic encephalopathy is a severe early‐onset epileptic encephalopathy with arrest of psychomotor development caused by hemizygous mutations in the ARX gene, which encodes a transcription factor in fundamental brain developmental processes. We presented a case report of 2‐year‐old boy, which indicates symptoms of Ohtahara syndrome.

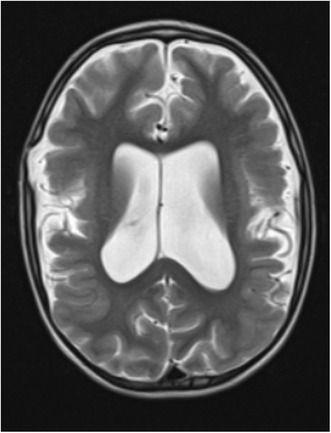

## 1 INTRODUCTION

### 1.1 Type 1 early infantile epileptic encephalopathy

Developmental and epileptic encephalopathies (DEEs) are characterized by the onset of refractory seizures in infancy or early childhood and are clinically and genetically heterogeneous. Individuals with DEEs have late or regressed psychomotor development, particularly after onset of seizures. DEE includes early infantile epileptic encephalopathies (EIEE) (Auvin et al., 2016). EIEE is a very early‐onset epileptic encephalopathy involving burst–suppression pattern on electroencephalogram (EEG) and suppression of psychomotor development (Jia et al., 2021). Also, it has been known as Ohtahara syndrome since Ohtahara (1976). The incidence of EIEE has been evaluated between 1 in 50,000 and 1 in 100,000 (Poke et al., 2022). EIEE is one of the age‐related epileptic encephalopathies in children (Ohtahara & Yamatogi, 2006; Yamatogi & Ohtahara, 2002). Type 1 EIEE (EIEE1) is a form of EIEE with an X‐linked recessive inheritance pattern. The cause of EIEE1 syndrome is hemizygous mutations in the Aristaless‐related homeobox (*ARX*) gene (https://www.omim.org/entry/300382) (Jia et al., 2021). Although male patients with *ARX* mutations are often more acutely affected, female carriers may also be affected (Kato et al., 2004; Wallerstein et al., 2008). But, the severity of the disease is higher in men than in women (Ekşioğlu et al., 2011).

In addition, EIEE is characterized by frequent seizures and poor prognosis. The treatment and prognosis of EIEE are challenging, with many cases being refractory to antiepileptic drugs. A study on EIEE caused by *SMC1A* gene truncating variation reported that the patients had severe developmental retardation, microcephaly, and refractory seizures, with poor response to treatment (Ye et al., 2022). Another article mentioned that EIEE is characterized by very early onset, frequent tonic spasms, and a suppression–burst pattern on EEG, with the course being severe and often leading to early death or marked psychomotor retardation (Radaelli et al., 2018). Additionally, a study on malignant migrating partial seizures of infancy, a rare early infantile epileptic encephalopathy, reported ineffective seizure control, severe retardation, and poor outcomes in some patients, highlighting the challenging prognosis of these conditions (Cai et al., 2021).

### 1.2 Symptoms

EIEE symptoms appear in the first 3 months of life, but some appear in the first few weeks after birth (Ohtahara, 1976). Patients with EIEE1 have variable types of seizures which start between birth and 3 months of age (Kato et al., 2004). Symptoms that patients illustrate include having poor suckling reflexes, hypotonia of the muscles, and manifest themselves with generalized and symmetrical tonic (Cheng et al., 2017). The pattern of occurrence of these spasms does not depend on the time of waking and sleeping, and the patient may experience these symptoms hundreds of times a day (Schlumberger, 1992). Other seizures that one‐third of patients may experience include hemic seizures, focal motor seizures, and generalized tonic–clonic seizures (Malik et al., 2013). If patients with EIEE1 survive after 2 years of age, they may experience more severe symptoms, including dyskinetic movements, an atypical Rett syndrome phenotype, West syndrome (WS), and Lennox–Gastaut syndrome (Ohtahara & Yamatogi, 2003; Yamatogi & Ohtahara, 2002). Patients may often experience death due to pneumonia or other complications of a complex disability (Ohtahara & Yamatogi, 2006; Radaelli et al., 2018).

EEGs demonstrate burst–suppression patterns in EIEE patients, and their global development is severely impaired; and they may die as early as infancy (Fullston et al., 2010; Ohtahara & Yamatogi, 2006). EIEE1 inheritance pattern is X‐linked recessive (Kato et al., 2010).

### 1.3 Diagnostic methods

There are various techniques to diagnose EIEE1. The EEG index within patients with EIEE1 illustrates a suppress–burst pattern (which appears with the onset of spasms). This pattern is constant throughout the day (Kato et al., 2010; Schlumberger, 1992).

Another method is to use differential diagnoses. In this method, an attempt is made to differentiate between EIEE1 and other epileptic encephalopathies such as early myoclonic encephalopathy, WS, and other early epileptic encephalopathies (Kato et al., 2010).

Genetic counseling and prenatal diagnosis are feasible in patients with EIEE1 and the cause of genetic disease has been distinguished (Bayat et al., 2021).

### 1.4 Management and treatment

Unfortunately, there is no known cure for this condition. However, there are some treatment options available that may help to manage and improve the symptoms of EIEE. Drug therapy is one such option, and it involves the use of antiepileptic drugs such as levetiracetam, valproate, phenobarbital, zonisamide, and benzodiazepines. These drugs work by reducing the number and severity of seizures experienced by the patient. Another treatment option is diet therapy, specifically the use of a ketogenic diet. This diet is high in fat and low in carbohydrates and has been shown to be effective in reducing the frequency and severity of seizures in some EIEE patients. Finally, in some cases, surgical intervention may be recommended. This involves the removal of the part of the brain accountable for the seizures, and can sometimes lead to a considerable improvement in the patient's symptoms. Despite these treatment options, patients with EIEE still need constant monitoring and care, as there is no known cure and the state can have significant long‐term effects on the patient's cognitive and neurological development (Beal et al., 2012; Clarke et al., 1987; Ishii et al., 2011; Komaki et al., 1999; Yamatogi & Ohtahara, 2002).

### 1.5 Prognosis

The prognosis for individuals suffering from EIEE is assumed to be poor. Patients often experience intense psychomotor disturbances that may manifest as developmental delays, intellectual disabilities, and motor impairments. Additionally, they may experience persistent seizures that can be difficult to manage. Unfortunately, the mortality rate for EIEE is high, with nearly 50% of individuals passing away before the age of 2. This highlights the urgent need for effective treatment options for EIEE to help improve the quality of life for affected individuals and to increase their chances of survival (Yamatogi & Ohtahara, 2002).

### 1.6 *ARX* gene

The *ARX* gene is able to regulate the activity of other genes (Kahrizi, 2012). This gene encodes a transcription factor, as a developmentally regulated homeobox transcription factor, which plays a crucial role in the brain growth process (Jia et al., 2021). The *ARX* gene is located in Xp22.13 in closeness to the 3′ end of POLA (DNA polymerase‐alpha) (Miura et al., 1997; Saitsu et al., 2008). In terms of positioning on the chromosome, the *ARX* and *POLA* genes are in tail‐to‐tail orientation. In addition, the *ARX* gene contains five coding exons and its mRNA length is approximately 12.5 kb (Kitamura et al., 2002; Strømme et al., 2002). This mRNA is translated into protein with 565 amino acids (Kahrizi, 2012). This encoded protein, as a homeoprotein, is classified into the paired (Prd) class. It includes a paired homeodomain, a C‐terminal domain (well known as OAR domain and C‐peptide), and a conserved octapeptide motif, which acts as transcriptional repressor (Banerjee‐Basu & Baxevanis, 2001; Gécz et al., 2006). The *ARX* gene has profound effect on differentiation and maintenance of certain neuronal cell types, especially within the central nervous system. Therefore, it plays a key role throughout vertebrate embryogenesis (McKenzie et al., 2007; Ohira et al., 2002; Poirier et al., 2004; Strømme et al., 2002). The *ARX* gene is expressed in the developing hypothalamus, pancreas, thalamus, forebrain, basal ganglia, and cerebral cortex; modulates migration and fate specification of interneurons; and regulates ventricular zone proliferation and testes (Marsh & Golden, n.d.; Gécz et al., 2006). Since the first report in 2002, *ARX* is related to infantile spasms, a spectrum of developmental matters, and syndromic and non‐syndromic forms of X‐linked mental retardation (XLMR) (Absoud et al., 2010; Wallerstein et al., 2008). Mutations in the *ARX* gene lead to various disorders such as X‐linked infantile spasm (ISSX), X‐linked myoclonic seizures, hydranencephaly with abnormal genitalia, X‐linked lissencephaly with abnormal genitalia (XLAG), spasticity and intellectual disability, Partington syndrome, nonsyndromic X‐linked intellectual disability, Ohtahara syndrome, idiopathic infantile epileptic dyskinetic encephalopathy, and Proud syndrome (Bienvenu et al., 2002; de Souza et al., 2006; Grønskov et al., 2004; Kato et al., 2004, 2007; Kitamura et al., 2002). Here, we describe a family with EIEE1 syndrome confirmed by a biochemical and genetic study, and finally, discuss the clinical, biochemical, and therapeutic aspects of the disorder (Table 1).

**TABLE 1:** Other studies have reported mutations in the *ARX* gene associated with EIEE1 disease.

	Our study	Absoud et al. (2010)	Arafat et al. (2017)	Ekşioğlu et al. (2011)	Fullston et al. (2010)	Giordano et al. (2010)	Shoubridge et al. (2019)	Tapie et al. (2017)
Mutation	c.84 C>A	c.333–334ins[GCG]7	c.1600G>C	c.98T>C	c.81C>G	c.1604T>A	c.1449‐1G>C	c.1616C>A
Consanguineous parents	+	−	−	−	−	−	−	−
Family history	+	−	+	+	+	+	+	+
Developmental retardation	Intrauterine developmental retardation	DD	DD	DD	DD	Growth deficiency	DD (delay of expressive and receptive language and social skills)	DD
Microcephalic	+	NR	NR	NR	NR	+	+	NR
Cataracts	+	NR	NR	NR	+	NR	NR	NR
Nystagmus	+	NR	NR	NR	NR	NR	NR	NR
Genital dysfunction	Small testes	Normal	NR	Ambiguous genitalia Micropenis Bilateral cryptorchidism	Normal	Normal	Normal	Ambiguous genitalia
Dysmyelination	+	+	Normal	NR	‌ Normal	NR	+	NR
Spastic quadriplegia	+	NR	NR	NR	NR	NR	+	NR
Seizures	+	+	+	+	+	+	+	+
Electroencephalogram pattern	HAR Severe multifocal epileptic abnormalities	SB	Intermittent SB during sleep cycle	SB	HAR	HAR and SB	Normal	HAR and SB
Hematologic test	Megaloblastic anemia Thrombocytopenia	Normal	NR	Normal	Normal	Normal	NR	Normal
Cerebrospinal fluid	Decreased serine and glycine levels	Normal	NR	Normal	NR	Normal	NR	Normal
Others	Adduction of the thumbs Reduced activity of 3‐PHGDH Low concentrations of fasting serine and glycine	Dyskinetic movement disorder Unresponsive to stimulation	Intellectual disabilities	Eyes rolling up Whole body extension of all extremities Occasional episodes of swimming movements Abrupt laughing Hypotonia Hyperreflexia Ankle clonus bilaterally	Gallstones Unable to feed oral	Intellectual impairment Hypotonic	Mild hypotonia Gross motor Mild plagiocephaly	−

Abbreviations: −: negative; +: positive; DD, developmental delay; HAR, hypsarrhythmia; NR, not reported; SB, suppression–burst.

### 1.7 Patient and observations

#### 1.7.1 Materials and methods

##### Ethical compliance

The study conducted at Shahid Sadoughi University of Medical Sciences in Yazd, Iran, was approved by the Ethics Committee, specifically the IR.SSU.SPH.REC.1400.005. The parents of the proband participated in the study provided written informed consent, indicating their willingness to get involved and their understanding of the study's purpose and potential outcomes. This agreement and consent illustrate the ethical considerations and procedures followed by the university in conducting the study. By obtaining consent and adhering to ethical guidelines, the university ensures that the rights and well‐being of the participants are protected.

### 1.8 Case presentation and results

**FIGURE 1:**
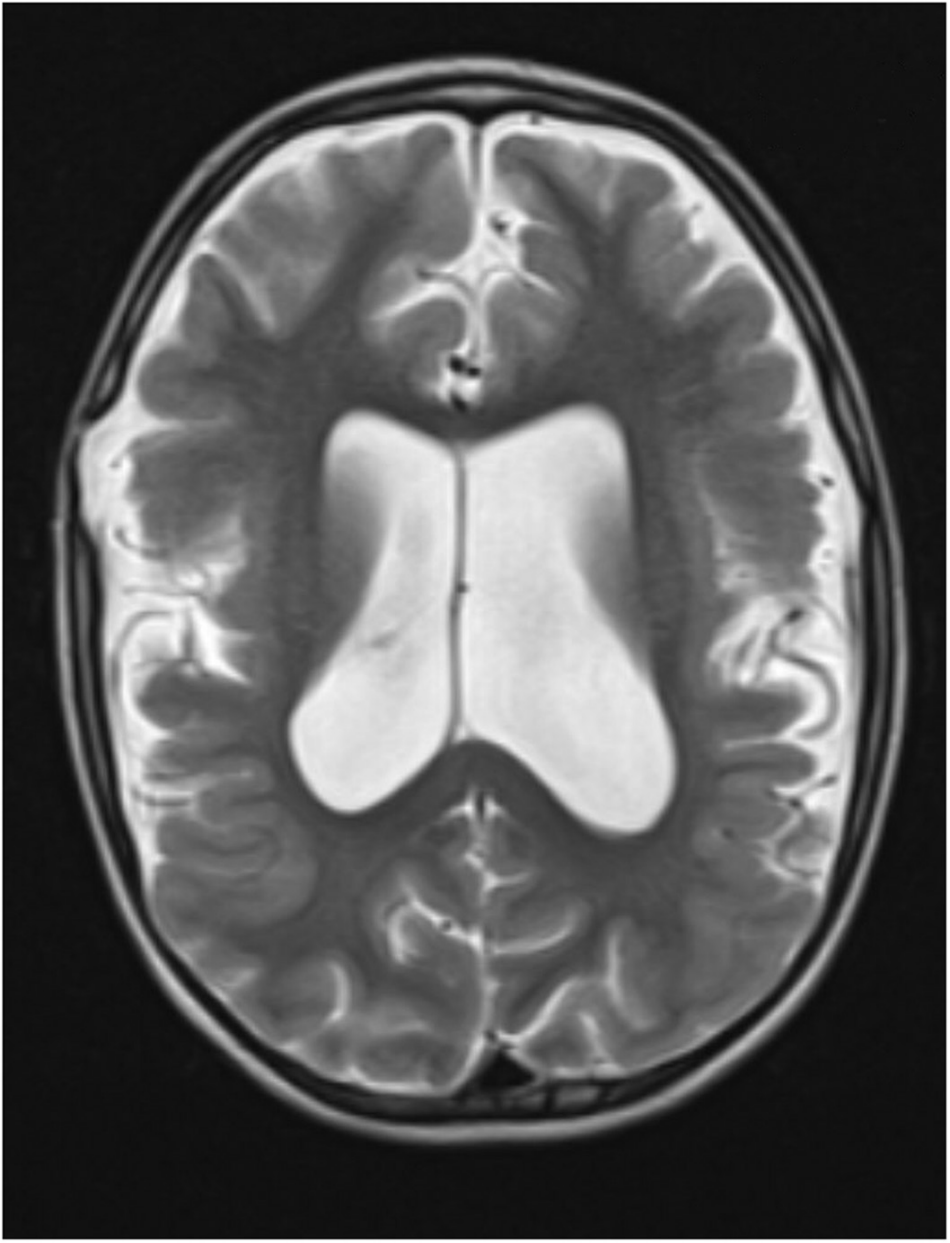
Magnetic resonance imaging of patients suffering from dysmyelination and seizures.

A family history of similar illness was present in the third‐degree healthy consanguineous Iranian parents who had three children: two boys who were patients and a healthy girl. The first boy was born patient and unfortunately passed away. The second boy had a complex pregnancy due to intrauterine growth retardation and was born with microcephaly and cataracts. Additionally, he was recognized with nystagmus, adduction of the thumbs, and small testes. At 2 years old, this boy was suffering from dysmyelination, spastic quadriplegia, and seizures. The EEG showed a hypsarrhythmia pattern or acute multifocal epileptic abnormalities (Figure 1). His hematologic test showed megaloblastic anemia and thrombocytopenia, while plasma amino acid analysis manifested low fasting concentrations of serine and glycine. Further research of his cerebrospinal fluid (CSF) showed decreased levels of serine and glycine. Enzymatic analysis of 3‐*PHGDH* (NC_000001.11) activity was measured in cultured skin fibroblasts, and it was found that the activity was decreased.

### 1.9 Molecular analysis

The researchers obtained informed consent from the parents and collected peripheral blood samples from both parents and their affected second child for DNA analysis. The study utilized three methods to analyze mutations including autozygosity mapping, Sanger sequencing, and whole‐exome sequencing. The first method used was autozygosity mapping, which recognized two regions of the genome with homozygous mutations. The first region was located on chromosome 8 and spanned from positions 23,551,904 to 37,671,140, covering a distance of 14 megabases. The second region was located on chromosome 1 and spanned from positions 120,188,180 to 145,588,184, covering a distance of 25.5 megabases. After identifying these candidate regions, Sanger sequencing and whole‐exome sequencing were performed to identify specific mutations within these regions.

**FIGURE 2:**
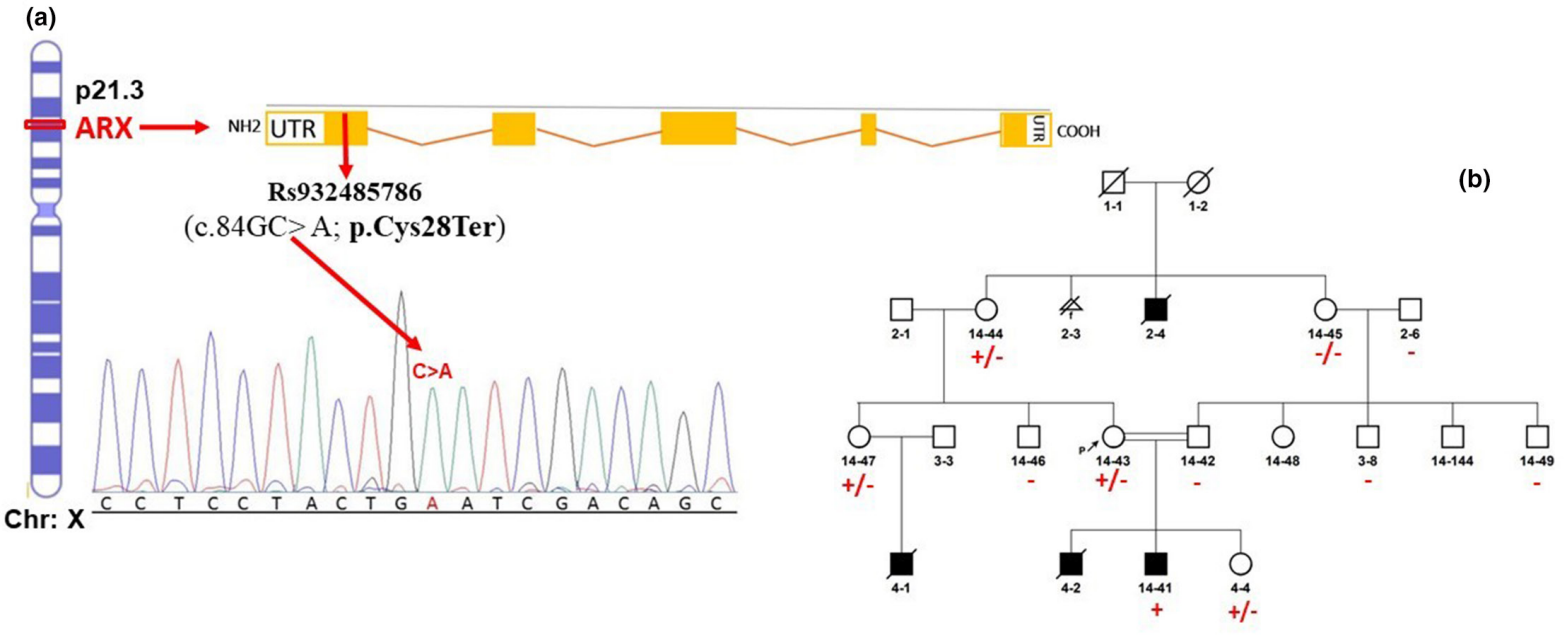
(a) Sequence analysis of ARX gene revealed p.Cys28Ter mutation. (b) Pedigree shows family members with EIEE1 phenotype (in black) with segregation of the p.(Cys28Ter) variant in ARX gene. Squares represent males; circles, females; +/−, heterozygote for p.(Cys28Ter) variant; and +/+ homozygous for p.(Cys28Ter) variant.

In this study, the researchers used autozygosity mapping and Sanger sequencing for mutation analysis of four candidate genes, including *TTI*, *ERLIN*, and *ENFL* in the chr8: 23,551,904 and 37,671,140, but did not find any mutations. Also, first, we used two methods, autozygosity mapping and Sanger sequencing, for mutation analysis of one candidate gene, including *PHGDH* in the chr1: 120,188,180–145,588,184, but did not find any mutations. Therefore, the researchers used the whole‐exome sequencing technique for mutation analysis in the *ARX* gene (NC_000023.11). Finally, mutation analysis, with whole‐exome sequencing technique, of the *ARX* gene confirmed a homozygous stop gained mutation (NM_139058.3:c.84C>A; p.(Cys28Ter)) (Figure 2). Both parents were shown to be carriers of the mutation confirming X‐linked recessive inheritance.

## 2 DISCUSSION

EIEE1 (Ohtahara syndrome) is a rare X‐linked recessive disorder specified by acute early‐onset epileptic encephalopathy with the stop of psychomotor development, which arises from the mutations in the *ARX* gene (Scheffer et al., 2017; Shoubridge et al., 2010). *ARX* includes five exons encoding a 562 residues protein, containing a paired‐class homeodomain and four repeats of 7–16 alanine residues called “polyalanine tracts” (Kitamura et al., 2002; Strømme et al., 2002). Polyalanine tract expansions and nonconservative missense mutations are milder genetic alterations outside of functionally principal domains that result in clinically less severe phenotypes such as XLMR, Partington syndrome, and ISSXs syndrome (Kato et al., 2004; Sherr, 2003; Shoubridge et al., 2010). *ARX* is expressed in GABAergic neurons, and dysfunction of the GABAergic system is vital for EIEE neuropathology and WS (Kato et al., 2007).

The main feature of the mutation phenotype in *ARX* is intellectual disability. This phenotype can be seen as the only clinical feature or frequent with extra symptoms, such as epilepsy, corpus callosum agenesis, genital abnormalities, lysencephaly, hydrocephalus, neonatal spasm, autism, dysarthria, or dystonia (Hartmann et al., 2004; Shoubridge et al., 2010).

Despite years of investigation, many aspects of *ARX* action in EIEE1 remain unknown due to the absence of a perfect in vitro disease model (Jia et al., 2021). Here, we demonstrate that NM_139058.3:c.84C>A; p.(Cys28Ter) (p.Cys28Ter), a variant within exon 1 of the *ARX* gene, can cause EIEE1. NM_139058.3:c.84C>A; p.(Cys28Ter) is a homozygous stop‐gained mutation and a loss‐of‐function variant that can impact the protein. We identified NM_139058.3:c.84C>A; p.(Cys28Ter) in a proband with early‐onset developmental and epileptic encephalopathy and his mother. Our patient had familiar prenatal backgrounds. All reported cases of pathogenic variants in *ARX* lead to phenotypes of seizure (Arafat et al., 2017; Bettella et al., 2013; Conti et al., 2011; Ekşioğlu et al., 2011; Fullston et al., 2010, 2011; Giordano et al., 2010; Kato et al., 2010; Kwong et al., 2015; Sartori et al., 2011; Strømme et al., 2002; Tapie et al., 2017). Both patients had a hypsarrhythmia pattern or severe multifocal epileptic abnormalities (Milh et al., 2011). Eified by early onset of tonic spasms, a characteristic suppression–burst pattern on the EEG, seizure intractability, and poor outcome with severe psychomotor retardation (Djukic et al., 2006; Ohtahara & Yamatogi, 2006). A study examined the *ARX* gene mutation in a patient‐reported ambiguous genitalia and global developmental delay phenotypes with poor cognitive abilities (Sirisena et al., 2014). In line with the previously reported *ARX* phenotypes described in scientific articles (Ekşioğlu et al., 2011; Hartmann et al., 2004; Ogata et al., 2000; Uyanik et al., 2003), EIEE can be caused by mutations in protein homeodomains because mutations outside the functionally important DNA‐binding motif of the gene, as mentioned, cause milder symptoms. It is hypothesized that the genital ambiguity in *ARX* mutations is mostly due to defective testis formation (Ogata et al., 2000). A similar study was conducted by Fullston et al. (2010). Detected a shortening mutation in the *ARX* gene (Y27X) in two patients with a clinical diagnosis of Otahara syndrome. Patients had a severe form of Otahara syndrome with refractory seizures. However, it does not cause X‐linked lysencephaly associated with ambiguous genital syndrome (XLAG). Overexpression of this mutation in HEK293 cells indicated the presence of a truncated *ARX* protein at the N‐terminal possibly using the start codon at residue 41. Because null *ARX* mutations are commonly associated with lysencephaly and ambiguous genitals, the *ARX* protein appears to be partially functional by resuming mRNA translation in these patients. A bioinformatics analysis was performed by Mustafa et al. (2020) to investigate SNPs' functional and structural implications in the human *ARX* gene associated with EIEE1. The analysis results showed the presence of 11 mutations (G34R, R528S, L33P, V374D, L343Q, L535Q, T333S, R332H, R330H, T333N, and R380L), which significantly altered the structure of the human *ARX* protein, which may disrupt the domain and affect protein function. In the meantime, the Cys28Ter mutation has not been considered. Despite the fatal symptoms seen in this patient, it is still believed to be a pathogen‐like mutation. Although these findings widen the spectrum of clinical phenotypes caused by mutations in the *ARX* gene, they also emphasize the molecular pathogenetic effect of individual mutations and the impact of genetic background resulting in intrafamilial clinical heterogeneity for these mutations. Since it is observed in the patient's laboratory symptoms, CSF serine and glycine levels were decreased, and enzymatic analysis of 3‐*PHGDH* activity was measured in cultured skin fibroblasts and showed decreased activity. These symptoms were compared with the symptoms mentioned in https://www.omim.org/entry/308350 in the field of this disease, and no case was found, so it can be reported as a new symptom in this disease that deserves further discussion and study.

Actually, the decreased activity of 3‐PHGDH, an enzyme involved in the serine synthesis pathway, presents a novel consideration in the context of EIEE1. While this feature is not a previously known characteristic of the condition, it prompts important inquiries into its potential relevance to the disease phenotype. The potential association between decreased 3‐*PHGDH* activity and EIEE1 warrants investigation, given the role of the serine synthesis pathway in neurological development and the emerging understanding of the impact of related genetic mutations on neurological function. Furthermore, it is plausible that a patient diagnosed with EIEE1 may also have an undetected second diagnosis of 3‐*PHGDH* deficiency due to underlying *PHGDH* mutations. This possibility highlights the complexity of genetic interactions and the importance of comprehensive genetic screening in individuals with neurological disorders (Noh et al., 2012).

## 3 CONCLUSION

Early‐onset epileptic encephalopathies represent a significant challenge in neurological clinical practice. In this scenario, genetic epileptic encephalopathies play a central role. In this case study, we present a patient suffering from EIEE1. Microcephaly, cataracts, nystagmus, adduction of the thumbs, and small testes were the physical symptoms of the patient. Electroencephalography results showed a hypsarrhythmia pattern or severe multifocal epileptic abnormalities. Dysmyelination, spastic quadriplegia, and seizures were also reported. Genetic studies showed significant results that the NM_139058.3:c.84C>A; p.(Cys28Ter) mutation in the *ARX* gene could be one of the factors involved in developing EIEE1 disease. With these interpretations, genetic testing can be considered one of the essential methods of diagnosing EIEE1.

## AUTHOR CONTRIBUTIONS

Erfan Zaker and Negar Nouri interpreted the results, conceived the idea, revised the literature, and wrote the manuscript; Mojtaba Movahedinia performed clinical assessment; Mohammad Yahya Vahidi Mehrjardi performed genetic analysis; and Ali Dadbinpour performed a critical revision of the manuscript for important intellectual content. All the authors have read and approved the manuscript.

## FUNDING INFORMATION

For this research, the author(s) did not receive any funding.

## CONFLICT OF INTEREST STATEMENT

The authors declare that they have no competing interests.

## ETHICS STATEMENT

This research was approved by the Yazd University of Medical Sciences Local Ethics Committee. The committee's reference number is IR.SSU.SPH.REC.1400.005.

## PATIENT CONSENT STATEMENT

Written consent was received from legal guardian of the patient.

## Data Availability

The data that support the findings of this study are available on request from the corresponding author.
